# Leukocyte Immunoglobulin-Like Receptors in Regulating the Immune Response in Infectious Diseases: A Window of Opportunity to Pathogen Persistence and a Sound Target in Therapeutics

**DOI:** 10.3389/fimmu.2021.717998

**Published:** 2021-09-14

**Authors:** Florence Abdallah, Sixtine Coindre, Margaux Gardet, Florian Meurisse, Abderrahim Naji, Narufumi Suganuma, Laurent Abi-Rached, Olivier Lambotte, Benoit Favier

**Affiliations:** ^1^Center for Immunology of Viral, Auto-Immune, Hematological and Bacterial Diseases (IMVA-HB/IDMIT), Université Paris-Saclay, Inserm, CEA, Fontenay−aux−Roses, France; ^2^Department of Environmental Medicine, Cooperative Medicine Unit, Research and Education Faculty, Medicine Science Cluster, Kochi Medical School, Kochi University, Nankoku-City, Japan; ^3^Aix-Marseille University, IRD, APHM, MEPHI, IHU Mediterranean Infection, SNC5039 CNRS, Marseille, France; ^4^SNC5039 CNRS, Marseille, France; ^5^Public-Hospital Assistance of Paris, Department of Internal Medicine and Clinical Immunology, Paris-Saclay University Hospital Group, Bicêtre Hospital, Le Kremlin-Bicêtre, France

**Keywords:** Leukocyte immunoglobulin-like receptors, immune evasion, immune checkpoint, infectious diseases, auto-immune diseases, virus, parasite, bacteria

## Abstract

Immunoregulatory receptors are essential for orchestrating an immune response as well as appropriate inflammation in infectious and non-communicable diseases. Among them, leukocyte immunoglobulin-like receptors (LILRs) consist of activating and inhibitory receptors that play an important role in regulating immune responses modulating the course of disease progression. On the one hand, inhibitory LILRs constitute a safe-guard system that mitigates the inflammatory response, allowing a prompt return to immune homeostasis. On the other hand, because of their unique capacity to attenuate immune responses, pathogens use inhibitory LILRs to evade immune recognition, thus facilitating their persistence within the host. Conversely, the engagement of activating LILRs triggers immune responses and the production of inflammatory mediators to fight microbes. However, their heightened activation could lead to an exacerbated immune response and persistent inflammation with major consequences on disease outcome and autoimmune disorders. Here, we review the genetic organisation, structure and ligands of LILRs as well as their role in regulating the immune response and inflammation. We also discuss the LILR-based strategies that pathogens use to evade immune responses. A better understanding of the contribution of LILRs to host–pathogen interactions is essential to define appropriate treatments to counteract the severity and/or persistence of pathogens in acute and chronic infectious diseases lacking efficient treatments.

## Introduction

The first line of defence against invading pathogen and host injury is the onset of an adequate inflammatory response. Its purpose is to mobilize effector cells and mediators of the immune system to prevent infection while promoting the repair of damaged tissues and reinstatement of immune homeostasis. Although a balanced inflammatory response is required to establish homeostasis, in some instances, infectious pathogens can induce a dysregulation of the immune response, thus resulting in an inappropriate inflammatory response and disease progression (1, 2).

The evolution from a beneficial to a harmful inflammatory response mostly depends on immunoregulatory receptors (3). In this regard, the family of leukocyte immunoglobulin (Ig)-like receptors (LILRs) include inhibitory (LILRB) and activation (LILRA) receptors that play a major role in regulating immune responses and inflammatory processes associated with the control or progression of infectious diseases (4). Indeed, LILRs coordinate the process of inflammation by stimulating or inhibiting immune-cell effector functions that include 1) cell migration, 2) cell proliferation, 3) phagocytosis, 4) cytokine production and secretion, 5) chemical mediator production and secretion and 6) cell death (5).

However, various infectious pathogens including human immunodeficiency virus (HIV), dengue virus, human cytomegalovirus (HCMV), *Mycobacterium tuberculosis*, or *Plasmodium falciparum* have evolved to subvert immunity by targeting LILR functions. In this regard, pathogens can directly produce molecules that bind to specific LILRs or indirectly modulate LILR expression. Herein, we provide a concise overview of the current knowledge of the 1) LILR multigene family members, 2) their cellular distribution from ligand binding to downstream signaling and 3) their immune functions in infectious diseases and beyond. Finally, we emphasize a cutting-edge understanding of how pathogens may take advantage of inhibitory LILRs to evade the immune response, thus leading to disease emergence.

## LILR Gene Organization and Plasticity

**Figure 1:**
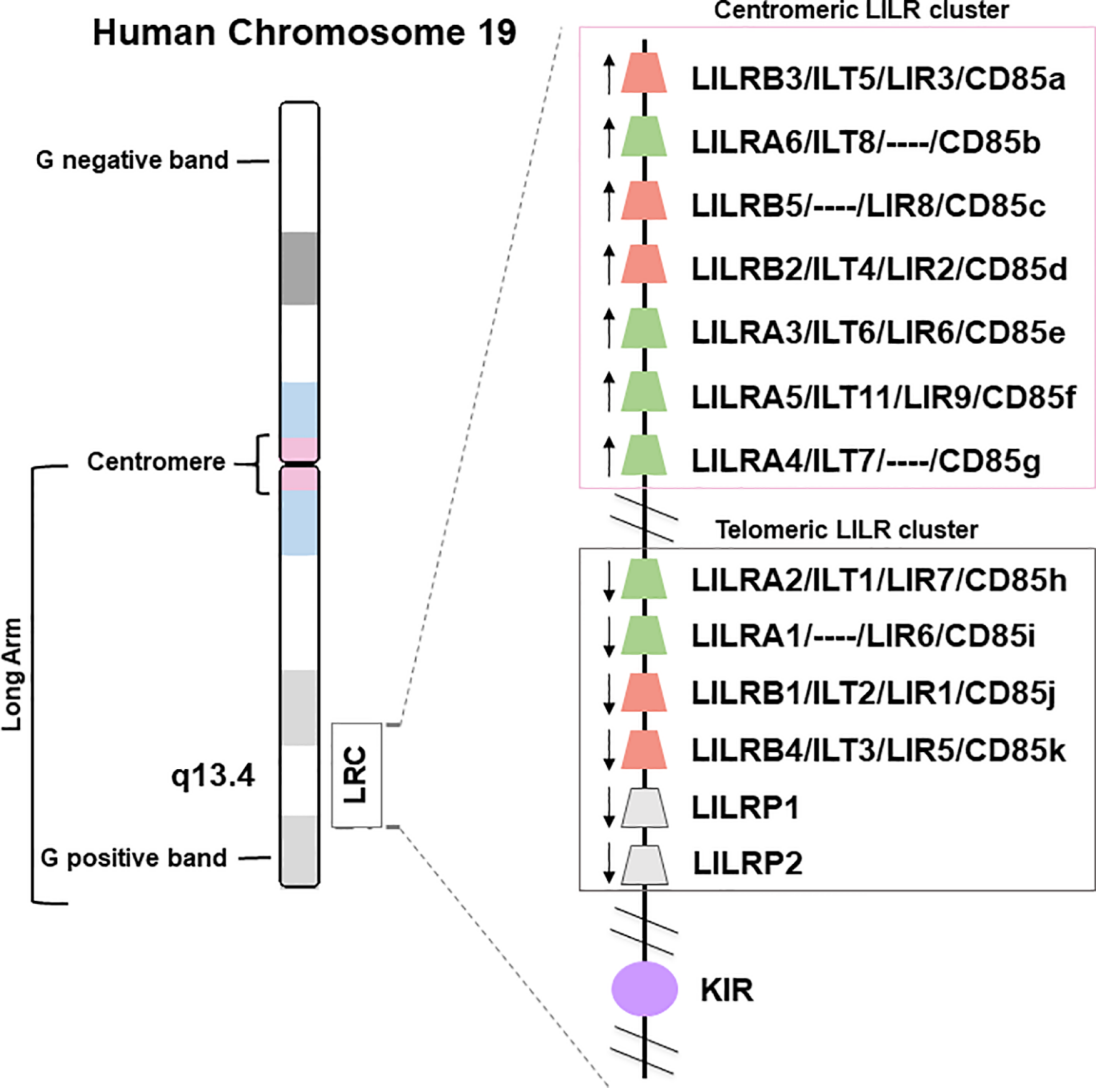
Genomic organization of the LILR gene cluster. The leukocyte immunoglobulin-like receptor (LILR) gene family is located on human chromosome 19q13.4, within the leukocyte receptor complex (LRC). The thirteen LILR genes are distributed into a centromeric and a telomeric cluster with opposite transcription orientations (→). A, activating (green); B, inhibitory (red); P, pseudogenes (grey); KIR, killer-cell immunoglobulin-like receptor (purple).

The *LILR* genes are all located on chromosome 19q13.4 in the Leukocyte Receptor Complex (LRC), a genomic region containing several other multigenic families of the innate immune system that belong to the Ig superfamily, such as the killer Ig-like receptors (KIR), the leukocyte-associated Ig-like receptors (LAIRs), and the sialic acid-binding Ig-type lectins (SIGLECs) (**Figure 1**). LILRs can encode inhibitory or activating receptors (5), and five LILR of each type are characterized: inhibitory receptors use the “B” letter in their names and include LILRB1-B5, while activating receptors use the “A” letter and include LILRA1-A2 and LILRA4-A6. LILRA3 is an exception as it is constitutively soluble due to a lack of exons encoding the transmembrane and cytoplasmic domains (6). Finally, *LILR* pseudogenes are assigned a “P” letter and include two genes: *LIRP1-P2* (7). These thirteen *LILR* genes are distributed into a centromeric (*LILRA3-A6*, *LILRB2-B3*, *-B5*) and a telomeric (*LILRA1-A2*, *LILRB1*, *-B4*, *LILRP1-P2*) cluster that have opposite transcription orientation and are separated by a cluster of *non-LILR* genes (**Figure 1**) (8, 9).

While activating and inhibitory LILRs are both highly related at the sequence level (10, 11) and structurally on their extracellular part, with two to four C2-type Ig-like domains, they notably differ in the structure and sequence of the linker domains, transmembrane domain, cytoplasmic tail, and 3’UTR region (12). Indeed, LILRBs possess a long cytoplasmic tail with immunoreceptor tyrosine-based inhibition motifs (ITIM- I/V/L/S-X-Y-X-X-L/V; X = any amino acid) (13, 14). In contrast, LILRAs have a shorter cytoplasmic tail with a positively charged residue (arginine) in the transmembrane domain: the charged residue enables association with an adaptor molecule, the FcRγ chain, which contains immunoreceptor tyrosine-based activation motifs (ITAMs) (10, 15, 16). This combination of structural similarity in the extracellular part and opposite signalling functions for the inhibitory and activating LILR is compatible with an evolution in response to pathogen pressure (17), as was proposed for activating and inhibitory KIR (18).

Consistent with a pathogen-driven evolution, gene families of the LRC such as KIR display a tremendous plasticity between closely related species such as human and chimpanzees (19). Although the same comparison showed a conserved genomic organization for the LILRs, it also revealed evidence of *LILR* gene polymorphism (20). Consistent with this, an increasing number of *LILR* alleles are being described in human populations: 11 for *LILRA3*, 14 for *LILRB3*, and 11 for *LILRA6* (21, 22). This sequence variation can also combine with alternative splicing to create more functional plasticity. Indeed, *LILRs* are highly susceptible to alternative splicing that can create variants that are either membrane-associated or putative soluble LILR (6, 21, 23). This phenomenon is increased in the context of tumors such as melanoma and colorectal and pancreatic adenocarcinoma, which are characterized by soluble LILR overproduction (6, 24).

Another form of plasticity of the LILRs is copy number variation (CNV), a phenomenon described for *LILRA3*, *LILRB3*, and *LILRA6* (22, 25). *LILRB3* and *LILRA6* exhibit CNVs that can be due to sequence deletion, non-allelic homologous recombination or crossing-over between non-allelic sequences. These CNVs positively correlate with mRNA and protein levels and lead to signaling modulation. Of note, despite the high degree of sequence identity between *LILRA6* and *LILRB3*, only the former exhibits CNVs that are positively correlated with the relative *LILRA6*/*LILRB3* mRNA ratio. Hence *LILRA6* CNVs can alter the expression of *LILRB3* on the myelomonocytic cell surface (25). In this manner, the biological outcome of LILRB3 engagement is compromised because of an LILRA6 CNV that disturbs the activating versus inhibitory signaling ratio (25). While genetic analysis of *LILRB3* highlighted high levels of non-synonymous variation and non-allelic homologous recombination, plasticity of *LILRA6* is even more marked and, in 20 of 48 human cell lines from the International Histocompatibility Working Group, *LILRA6* showed deletion or duplication at exons coding for the extracellular domains (exons 3 to 6) (22).

In addition to *LILRB3* and *LILRA6*, *LILRA3* also displays CNV and gene frequency of functional *LILRA3* differs across populations. Indeed, some individuals have a deficient *LILRA3* because of a large deletion that encompasses the exons coding for the leader peptide and Ig domains (26). This deletion can reach an allele frequency of more than 80% in North-East Asia (maximum of 84% in Korea) but is less common in Europe (~17%) and Africa (~7%) (27). Additionally, a *LILRA3* allele that is non-functional due to a premature stop codon is also prevalent in the same regions of the world, suggesting that *LILRA3* is a target of natural selection in these populations (27, 28).

Ongoing efforts from the scientific community are thus starting to address the extent and functional impact of the diversity of the LILRs, in particular in regard to their involvement in regulating inflammation and susceptibility to disease. Investigations of the impact of this *LILR* diversity on ligand-binding properties are also ongoing and promise to offer a better understanding of the contribution of natural selection, driven by host–pathogen interactions, to *LILR* plasticity. Hereafter, we give an overview of the growing list of LILR ligands.

## LILR Endogenous and Pathogen-Derived Ligands

More than 2 decades after the LILR discovery, the identification of their ligands was relatively slow. Some LILRs can recognize several heterogeneous ligands, but others remain as orphan receptors. The multimeric aspects of LILRs can result in several conformational rearrangements, hinge effects, and domain configurations, promoting large-scale binding to multiple ligands with diverse molecular composition. Moreover, in some pathological conditions, LILR structural properties are modified owing to polymorphism and alternative splicing. These modifications can change the receptor topology by inducing conformational fluctuations at the binding site that alter the nature and stability of the receptor–ligand interaction.

**Figure 2:**
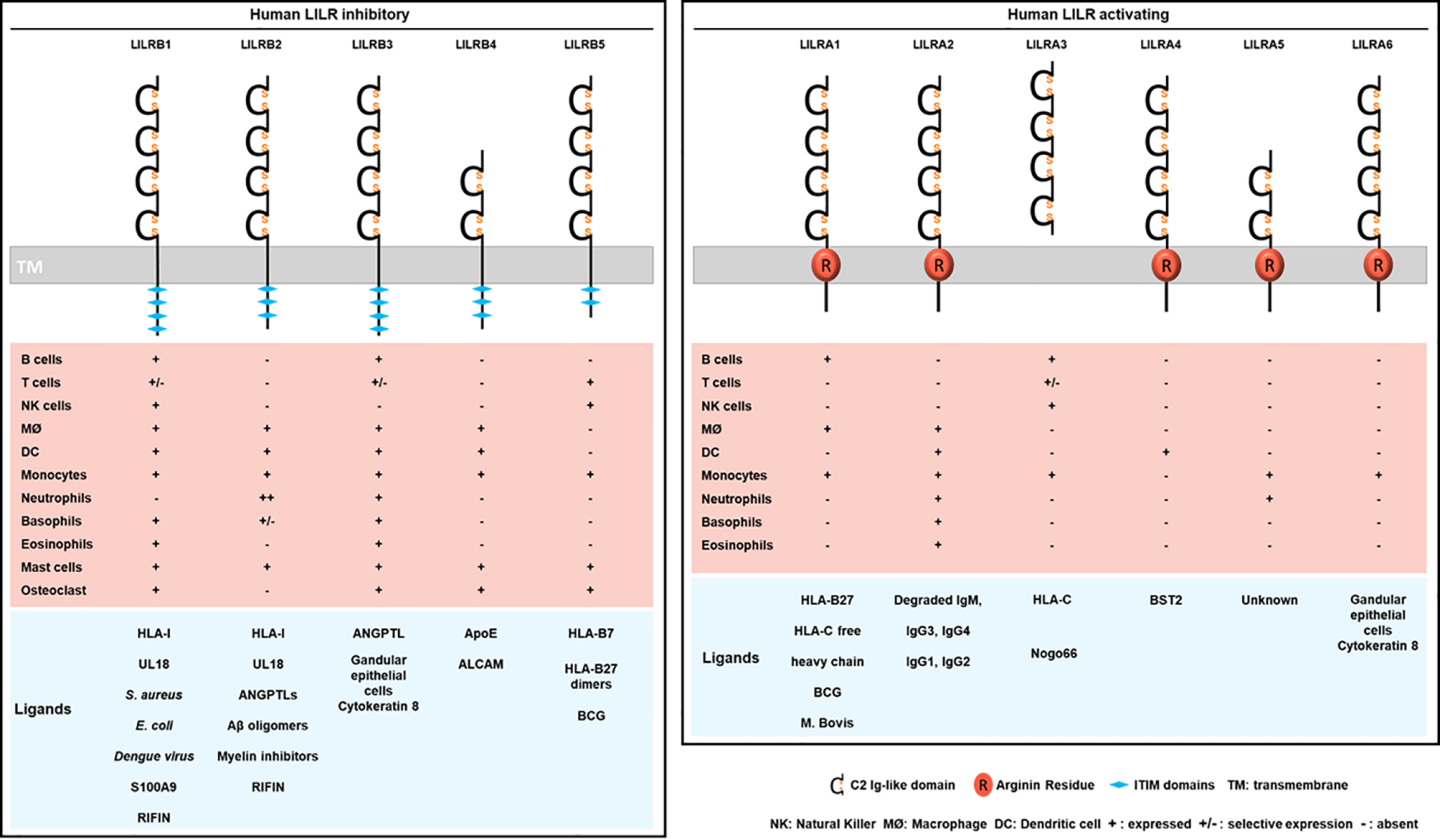
Structure, cellular distribution and ligands of the inhibitory LILRB (left) and activating LILRA (right). LILRB1-B5 are inhibitory LILRs that contain ITIM domains in their cytoplasmic part (left). LILRA1, LILRA2, LILRA4, LILRA5, LILRA6 are activating receptors with a short cytoplasmic tail and an arginine residue (in red) in their transmembrane portion (right). LILRA3 is soluble owing to the lack of a transmembrane and a cytoplasmic domain. Below is represented the LILR distribution on immune cell populations (MØ, macrophages). The different known ligands are shown in blue.

Some LILRs mainly recognize HLA-I ligands with various affinity according to HLA-I alleles but also to the amino acid sequence of the presented peptide (4, 29). Moreover, some LILRs are able to bind to HLA-I molecules that are dissociated from beta-2-microglobulin (B2M) (30). Because of the unique capacity of LILRs to regulate immune responses, some pathogens also produce ligands specifically targeting each LILR. This finding underlines the evolutionary plasticity of the LILR family and highlights the importance of selective pressure along with direct interaction with HLA-I ligands and evasion strategies of pathogens. In this section, we provide a global vision of the expanding spectrum of endogenous and exogenous ligands for LILR receptors (**Figure 2**).

### Native or Self-Ligands

Investigations identified LILRB1 and LILRB2 as receptors for a broad range of classical (HLA-A, HLA-B, HLA-C) and non-classical (HLA-F, HLA-G) HLA-I molecules (**Figure 2**) (29, 31). The ability of LILRs to bind this broad range of HLA-I ligands is supported by the conserved regions among HLA-I subtypes and alleles. In addition, this finding evokes the possibility of a parallel evolution of LILRs and their HLA-I ligands. However, the strength of the binding is influenced by several factors including the engaged HLA-I haplotype, the amino acid sequence of the presented antigenic peptide and LILR polymorphism (4, 26, 32, 33). Crystallography studies provided structural evidence for the binding domain of LILRB1 and LILRB2 to HLA-I, and LILR family members were then classified into 2 groups based on their sequence similarity, structural and biophysical characteristics of binding features with HLA-I molecules (11, 30, 34). Group 1 (LILRB1, LILRB2, LILRA1, LILRA2, and LILRA3) has highly conserved binding residues (amino acids) with an HLA-I contact region. In contrast, group 2 (LILRB3, LILRB4, LILRB5, LILRA4, LILRA5, and LILRA6) has poor conservation and was proposed to engage different set of ligands with a different binding mode (34).

The crystal structure of HLA-A2 bound to LILRB1 revealed the conservation of specific residues within the LILRB1 and LILRB2 first 2 Ig-like domains, D1-D2, at the interaction sites with HLA-I- B2M and α3 domains (34, 35). The crystal structure of LILRB2/HLA-G highlighted significant differences at the binding interface as compared with LILRB1/HLA-A2. LILRB2 recognizes HLA-I molecules by relying on a predominant interaction with the α3 domain independent of B2M. Similar observations were reported for LILRA1 and LILRA3 (29, 36). LILRB1 can only recognize HLA-I molecules associated with B2M, which indicates distinct recognition patterns between LILRB1 and LILRB2 (29, 37, 38). More recently, 2 alternative models have been proposed, including the 4 Immunoglobulin-like domains (D1-D2-D3-D4), and taking into consideration the impact of HLA-I polymorphisms as well as the sequence variations in the presented antigenic peptides. The models differ in the orientation and distance of α1 and α2 domains of HLA-I toward D1-D2-D3-D4 domains of LILRB1 and LILRB2, respectively (39). Studies demonstrated a direct interaction between D1-D2 and HLA-I. Surface plasmon resonance approach failed to show a substantial interaction between D3-D4 and HLA-I (31). Yet, recent evidence suggests a potential scaffold role of D3-D4: it ensures conformational changes by bending to allow the binding of D1-D2 with HLA-I *via* a *trans* interaction (34, 40, 41). In addition to this *trans* coupling, LILRB1 and LILRB2 can associate with HLA-I *via cis* interactions (37–39). This *cis* binding reduces receptor accessibility to a *trans* ligand interaction and thereby modulates the cellular activation threshold (40, 42). LILRA1 and LILRA3 also bind to HLA-I, but their interactions are weaker than their inhibitory counterpart. For instance, LILRA3 presents 1 or 2 amino acid changes in the binding region that results in reduced affinity toward HLA-I as compared with LILRB1 and LILRB2 (29, 36, 37). Like these two inhibitory receptors, LILRA1 was also reported to bind HLA-C and HLA-B27 (29, 37). As mentioned above, *in silico* analysis suggested that LILR group 2 members lack the required amino acid residues for interacting with HLA-I ligands (34). However, LILRB5 was reported to bind HLA-B7 and HLA-B27 free heavy chains (43).

### Pathogen-Derived or -Induced Ligands

**Figure 3:**
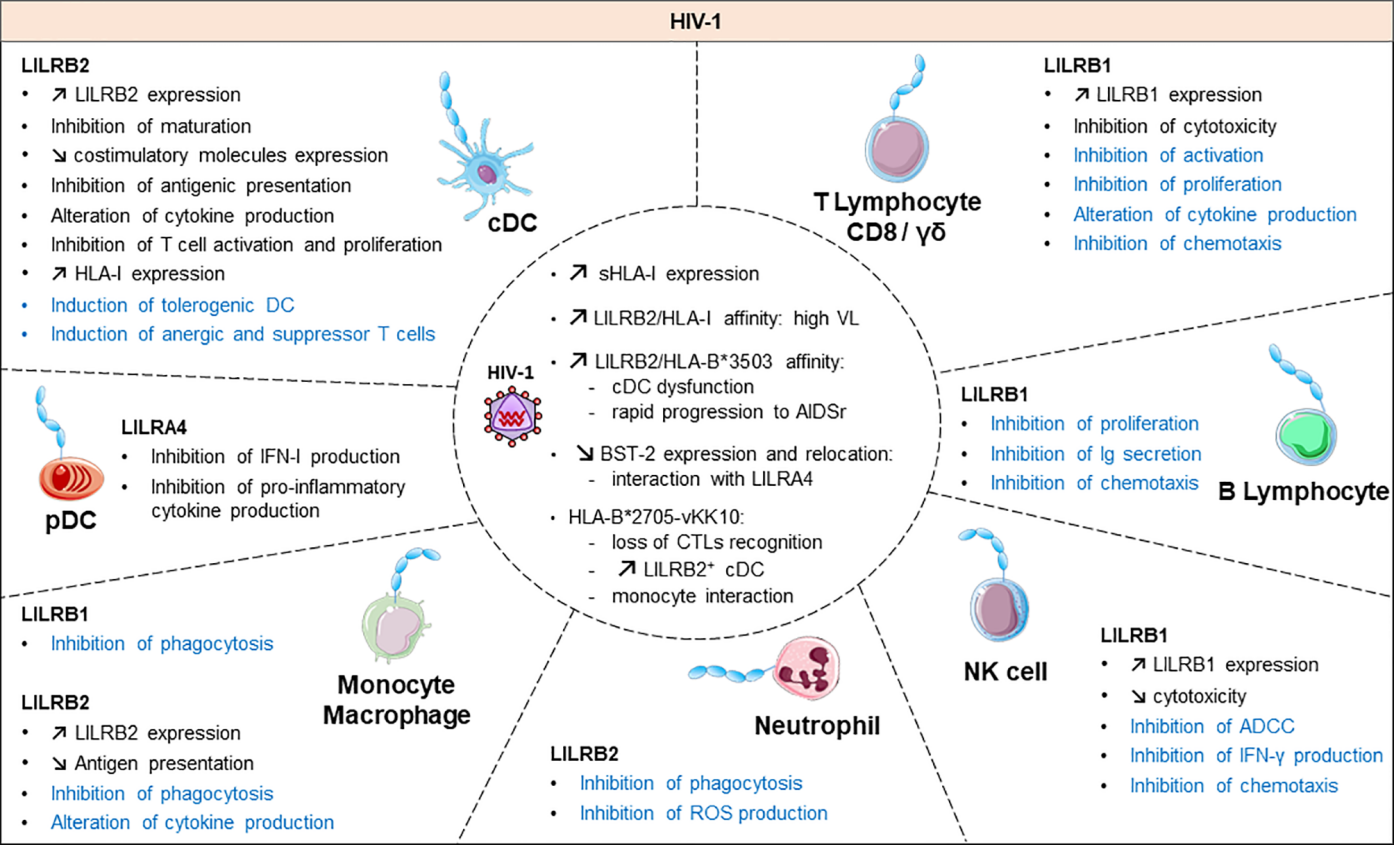
Functional roles and dynamics of LILRs in HIV infection. HIV infection induces a dysregulation of the immune response. In black, the direct impact of HIV in conventional dendritic cells (cDCs), plasmacytoid dendritic cells (pDCs), monocytes/macrophages, natural killer (NK) cells, CD8 T cells and γδ T cells are mentioned. The LILR-mediated dysregulation related to the upregulation of the HLA-I expression is shown in blue. ROS, reactive oxygen species; IFN-γ, interferon γ; Ig, immunoglobulin; ADCC, antibody-dependent cellular cytotoxicity; CTL, cytolytic T cell.

Besides recognizing HLA-I ligands, the LILR family can also recognize ligands derived directly from pathogens or *via* proteins from stressed cells (**Figure 3**). In 1997, Cosman and colleagues discovered that UL18, an HLA class I-related protein produced by human CMV, was a new ligand for LILRB1 (44). Then, LILRB2, but not other LILRs, was found to bind UL18 (23). Moreover, LILRB1 is reported to bind to dengue virus, *Staphylococcus aureus* and *Escherichia coli* (45, 46). However, molecules produced by these pathogens that bind to LILRB1 have not been characterized. Recently, LILRB1, was described to bind to repetitive interspersed families of polypeptides (RIFINs), which are produced by *P. falciparum* in infected erythrocytes (47). In addition, Sakoguchi and colleagues showed that a specific RIFIN could bind to LILRB2 with potential implications in immune evasion mechanisms of *P. falciparum* (48). Besides recognizing HLA-I or pathogen ligands, LILRB2 was found to bind with low affinity to oligomeric and monomeric synthetic amyloid-β (Aβ) (49) but strongly to Aβ42 oligomers *via* D1-D2 (50).

LILRB3 and LILRA6 can bind to a ligand from necrotic glandular epithelial cells associated with cytokeratin 8 (51). Nevertheless, further investigations are needed to clearly determine the identity of this ligand and the functional consequences of its interaction with LILRB3 or LILRA6. Cancer studies recently revealed 2 ligands of LILRB4: apolipoprotein E and CD166-activated leukocyte cell adhesion molecule (52, 53). LILRB5 could interact with Bacillus Calmette-Guérin, an attenuated pathogen of tuberculosis. This finding needs confirmation because it was achieved with a panel of 2B4 reporter cell lines for NFAT-GFP (54). Likewise, LILRA1 could preferentially bind Bacillus Calmette-Guérin over a weak interaction with *Mycobacterium bovis* (54). LILRA2 remained an orphan receptor until 2016 when Hirayasu and colleagues discovered accidentally, by *Mycoplasma hyorhinis* contamination of Daudi cells, that degraded IgM strongly associates with LILRA2 (55). This finding shed light on the LILRA2 recognition of distinct cleaved Ig by microbial proteases with stronger binding affinity to IgM, IgG3 and IgG4 than IgG1 and IgG2 (55). Several strategies including signal recognition particle, co-immunoprecipitation and biochemical assays were used to confirm a high-affinity specific and saturable binding of LILRA3 to Nogo 66 (potent inhibitor of axonal regeneration and neurite outgrowth after central nervous system injury) (56). LILRA4 recognizes the restriction factor human bone marrow stromal cell antigen 2 (BST2, also known as tetherin and CD317), which is upregulated in cancer and HIV-infected cells (57). To date, LILRA5 remains an orphan receptor (58).

Even though the discovery of LILR ligands is in constant progress, their characterization is challenging because these ligands present variable expression according to the tissue and physiological state. Moreover, some are produced only during infection by specific pathogens. The ability of certain microbial ligands to directly interact with LILRs provides opportunities for pathogens to orient the immune response in their favour. In response to the ligand association with LILRs, a cascade of conformational changes and downstream signalling is triggered. This situation results in a positive or negative signal depending on the type of engaged LILR. To date, the complete downstream signaling molecules remain poorly characterized. Nevertheless, biochemical studies indicate that ligand binding to LILRB induces a cascade of tyrosine phosphorylation, which provides a docking site for recruiting cytoplasmic phosphatases with Src homology 2 domain-containing protein tyrosine phosphatase 1 (SHP-1) and/or SHP-2 (14, 59). In contrast, LILRA molecules have a short cytoplasmic tail and deliver an activating signal by ITAMs within the FcRγ chain, which are associated with them. The phosphorylation of the ITAM domain, resulting from LILRA engagement, induces the recruitment and activation of tyrosine kinases such as Syk kinase in myeloid cells and B cells or ZAP-70 in lymphoid T cells (60, 61).

## LILR Distribution and Function

LILR members are mainly found on myeloid lineages such as monocytes, macrophages, neutrophils and dendritic cells (DCs). However, some LILRs are also found on B cells and subsets of natural killer (NK) and T cells. Because of their wide distribution on immune effector cells, LILRs are potent regulators of both the innate and adaptive immune response. Consequently, LILRs modulate an immune response by inducing or controlling inflammation. This section focuses on the current knowledge of LILR distribution and expression on immune cells and how these receptors shape the immune response under the physiological state. The LILR cellular distribution is summarized in **Figure 2**.

In the healthy condition, LILRB1 is expressed on various immune cell subsets including myeloïd cells (monocytes, macrophages and DCs), basophils, eosinophils, osteoclast precursors and mast cell progenitors. LILRB1 is also expressed on B cells and subpopulations of CD8, γδ T cells and NK cells (21, 42, 62, 63). Moreover, LILRB1 is specifically expressed on subsets of memory CD8 T cells and memory NK cells, which suggests a major role in regulating memory immune cytolytic cells (64–66). Functionally, the engagement of LILRB1 on CD8 T cells inhibits super-antigen–dependent cell cytotoxicity upon interaction with HLA-I in antigen-presenting cells (APCs) (21). LILRB1 engagement also impairs B-cell functions including proliferation and antibody production (21, 64, 67). In monocytes, LILRB1 specifically inhibits FcγRI-mediated phosphorylation as well as intracellular calcium mobilization (21, 65). In addition, LILRB1 has a central role in immune tolerance for self- *vs* non-self-signals. For instance, NK cells can distinguish normal healthy cells from abnormal cells *via* LILRB1 sensing HLA-I molecules. The binding of LILRB1 to HLA-I promotes inhibitory downstream signaling to prevent normal cells lysis by NK cells. The interaction of LILRB1 expressed on NK cells with HLA-G on target cells blocks lytic granule delivery and interferon γ (IFN-γ) production by impairing F-actin polymerization and microtubule-organization center polarization at the immunological synapse (66, 68). Of note, this inhibition of NK cell functions mediated by the LILRB1–HLA-G interaction is independent of lipid raft integrity on tumor cells (69). Furthermore, continuous stimulation of LILRB1 on DCs alters their differentiation programs and their capacity to produce cytokines even after exposure to lipopolysaccharide (70). Similarly, osteoclast differentiation inhibition was mediated by LILRB1 engagement and subsequent SHP-1 recruitment (71). Overall, LILRB1 fine-tunes major cellular and immunological processes by modulating the immune cell activation threshold.

LILRB2 is one of the most well-characterized receptors in the LILR family, with an important physiological role in various tissues because it interacts with ligands from different origins. Unlike LILRB1, LILRB2 is almost exclusively expressed on myeloid cells (42, 66, 68–70). It is well known for its tolerogenic effect in DCs that impairs CD4^+^ T cell activation (72, 73). Moreover, LILRB2-induced tolerogenic DCs can promote the induction of T regulatory cells (Tregs) (72, 73). A hallmark of LILRB2 is its ability to inhibit signaling pathways induced by different FcRs. In neutrophils, cross-linking FcγRIIa with LILRB2 abolishes the production of reactive oxygen species (ROS), whereas in DCs, it inhibits intracellular calcium mobilization (74–76). In addition, the co-aggregation of LILRB2 with FcγR *in vitro* inhibits monocyte-signaling events *via* SHP-1 recruitment (65). Similarly, LILRB2 transfection of RBL cells (basophilic-derived cell line) blocks serotonin secretion triggered by FcεRI (76). These data highlight the modulatory role of LILRB2 in immune responses induced by FcγR. The constitutive *cis* association between LILRB2 and HLA-I on basophilic cell lines (KU812) ensures self-recognition, so it prevents IgE-mediated allergic responses (42). In addition, LILRB2 engagement with angiopoietin like 2 (ANGPTL2) inhibits the differentiation but not proliferation of hematopoietic stem cells (HSCs) *ex vivo* resulting in their tumoral expansion (77, 78). Moreover, LILRB2 interferes with neural function by binding Nogo66 and inhibiting axonal regeneration (79). Similar to LILRB1, LILRB2 regulates osteoclastogenesis by inhibiting osteoclast differentiation *via* a *cis* interaction with HLA-I (71).

From cell-surface staining and RNA microarray analysis, LILRB3 expression was reported on monocytes and neutrophils, some T cells, osteoclasts and progenitor mast cells. However, the use of antibodies to detect LILRB3 remains challenging because of the high level of sequence homology of the extracellular fragments with LILRA6 (22, 25, 71, 76, 80, 81). LILRB3 function needs to be further characterized. Nevertheless, LILRB3 was demonstrated to antagonize FcεRI or LILRA2-mediated allergy in both IgE-dependent and -independent activation (82). In addition, LILRB3 triggering inhibits osteoclast differentiation, as do LILRB1 and LILRB2 (71). More recently, LILRB3 was reported to act on neutrophils as a potent inhibitor of Fc receptor–mediated effector functions, including ROS production, phagocytosis, and killing of microbes (83). Moreover, LILRB3 was found an important myeloid checkpoint receptor because of its immunosuppressive functions, which inhibit *in vitro* immune responses such as myeloid-induced T cell proliferation (84).

LILRB4 has only 2 extracellular Ig-like domains. Various cell types including myeloïd cells, progenitor mast cells, and osteoclasts express LILRB4 (71, 80) (23, 81, 85). LILRB4 blocks intracellular calcium mobilization in monocytes and macrophages upon co-ligation with CD11b, HLA-DR or FcγRIII (86). Similar to LILRB2, LILRB4 can induce immunosuppressive APCs (72, 73, 87).

LILRB4 dephosphorylates TRIM21, which results in Fc receptor-mediated cytokine production and inhibition of clathrin-dependent endocytosis in THP-1 cells (88). More recently, LILRB4 was found to exert dual regulation (positive or negative) of TNF-a production by THP-1 cells (89). LILRB4 was implicated in immune tolerance to allergens. An increased level of LILRB4 in CD4^+^CD25^+^FOXP3^+^ Tregs was associated with their failure to efficiently suppress allergen-specific T helper 2 (Th2) cell responses (90).

LILRB5 is expressed by a variety of immune cells including T cells, monocytes, NK cells, mast cells and osteoclasts (6, 23, 54, 80, 91). Yet, current knowledge about its function is limited. LILRB5 was first described as a membrane-associated molecule, but more recently a soluble form was detected (80). More precisely, LILRB5 is found in mast cell granules and is released to the extracellular milieu when these cells are activated (80). In addition, Hogan and colleagues proposed a role for LILRB5 in modulating a cytotoxic T-cell activation threshold (54). Thus, the identification of a LILRB5 natural ligand would motivate further investigations for better functional characterization and understanding of immune responses and inflammation.

Both LILRA1 and LILRA6 functions remain not well characterized. LILRA1 is found on monocytes, macrophages, and B cells (23, 25, 29, 80). The specific detection of LILRA6 is challenging because of its structural similarity with LILRB3 at the extracellular fragments. LILRA6 expression was demonstrated by RNA microarray analysis in monocytes, cultured macrophages, and osteoclasts (71). Nevertheless, the specificity of RNA microarray detection in this case remains questionable.

LILRA2 is preferentially expressed in the myeloid lineage (monocytes, DCs and macrophages), neutrophils, eosinophils and basophils, which suggests an important role in regulating the innate immune response (10, 11, 23, 82). LILRA2 can detect microbial immune evasion *via* its capacity to sense cleaved Igs by bacterial proteases (55). Yamazaki et al. provided recent insights into the molecular mechanism of LILRA2 recognizing the cleaved Ig that would occur *via* hydrophobic interaction of the LILRA2 D2 domain and exposed hydrophobic surface of N-truncated Ig (92). These findings highlight the molecular mechanisms of LILRA2-mediated inflammation to counteract the altered immune response induced by pathogens. Other studies show that in monocytes, LILRA2 engagement triggers calcium influx as well serotonin release, whereas in basophils, stimulation of LILRA2 results in increased allergic mediators such as histamine, leukotriene and interleukin 4 (IL-4) independent of IgE (82). These functional studies have unraveled the emerging role of LILRA2 as an important mediator of innate immune responses and inflammation involved in host defenses.

LILRA3 is a soluble receptor produced by monocytes, NK, B cells and some T-cell subsets (23, 26, 93). Although the immune functions of LILRA3 remain poorly characterized, it is involved in nervous system plasticity. In this context, LILRA3 acts as a decoy to Nogo66, a negative regulator of axonal regeneration. As well, LILRA3 might be a competitive antagonist with LILRB2 *via* high-affinity interaction with Nogo66 resulting in neurite outgrowth and synapse formation (56).

LILRA4 has a restricted cellular distribution as compared with the other LILR members. So far, it is uniquely found on plasmacytoid dendritic cells (pDCs) (94, 95). LILRA4 expression is variable depending on the state of pDC activation induced by viral or bacterial stimulation (96). Several pDC-like cell lines derived from CD4^+^CD56^+^ leukemia cells lack LILRA4 expression (96). Although it belongs to the activating family, LILRA4 was mainly reported to have inhibitory functions. In this regard, LILRA4 interacting with BST2 suppresses pDC activation, thus compromising the pDC production of type-I IFNs (57). This negative regulation is induced by the signal adaptor protein FcεRIγ, which forms a complex with LILRA4. The transduced ITAM-mediated signals negatively modulate Toll-like receptor (TLR)-induced type-I IFN production by human pDCs (95).

LILRA5 transcripts are found in several tissues of the hematopoietic system including bone marrow, spleen, lymph node, and peripheral leukocytes, although the protein form of LILRA5 is mostly expressed in monocytes and neutrophils (58, 97). More recently, proteomic studies identified LILRA5-specific peptides in neutrophils of patients with monogenic diseases (98). A soluble form was detected in transfected COS cells (97). In monocytes, LILRA5 stimulation results in intracellular calcium mobilization. Moreover, triggering LILRA5 in monocytes stimulates cytokine production implicated in early stages of inflammatory responses including IL-1β, tumor necrosis factor α (TNF-α) and IL-6, which suggests a modulatory function in inflammatory settings (97). The identification of LILRA5 ligands will be helpful to further delineate its function.

## The Interplay of LILRs With Infectious Pathogens and Inflammation

Given the major role of LILRs in regulating the immune response, several pathogens target their immunomodulatory functions to escape immunosurveillance. Modulation of LILR activity is directly linked to the inflammatory response induced after viral, bacterial or parasitic infection. In this context, LILRs behave as pathogenic mediators by compromising the immune response and associated inflammation.

### LILRs in Viral Infection

Emerging data have revealed the involvement of LILRs in different aspects of HIV-1 disease pathogenesis and progression. **Figure 3** summarizes the implication of LILR in HIV-1 pathogenesis. The LILR expression profile on immune cells varies between individuals who are HIV-positive in the acute phase, the chronic phase, under combination antiretroviral therapy (HIV cART^+^) or in elite controllers who naturally control HIV-1 infection, which suggests an interplay of these receptors in immune responses against HIV (99–102). Long considered a T-cell disease, HIV disease progression is considered to originate from a complex cross-talk between various immune cells, implicating both innate and adaptive immunities. HIV-induced immune activation has double-edged consequences on disease evolution. It first fights the virus by increasing immune-cell mobilization and modulating immunoregulatory receptors such as LILRs or their ligands, including HLA-I molecules or BST2 (4). However, these defence strategies induce concomitant inflammatory and immunosuppressive responses that can participate in viral persistence. Several studies have provided insights into the functional role of LILR repertoires and HLA-I polymorphisms in HIV disease progression. The framework orientation toward LILR function in HIV-induced pathology started with growing evidence that pointed to a correlation between HLA-I genotype variants and the rate of acquired Immunodeficiency syndrome (AIDS) development (103, 104). In particular, HLA-B27 and B57 were found associated with slow progression and HLA-B35 with rapid progression (103). These results were reinforced by targeted genotyping and whole genome analysis of different clinical cohorts that revealed an important role of the HLA-C locus in HIV disease control (105–110). These HLA-I polymorphisms have a great impact on the binding affinity toward LILRs. Indeed, besides their impact on antigen presentation to cytotoxic T lymphocytes, our understanding of how these HLA-I genetic variants modulate immune responses and HIV progression emerged only a few years ago. Investigations into the role of LILRs in HIV have foreshadowed a functional significance of the binding between HLA-I and LILR molecules during HIV pathogenesis, which is associated with viral replication and immune dysregulation. Hence, this immunomodulatory function of HLA-I mediated by LILR balances the immune response and influences the clinical HIV-1 infection outcome. Ten years ago, Huang and colleagues demonstrated a compromised *ex vivo* DC function in HIV-1–infected carriers as a result of a selective interaction between LILRB2 and the HLA-B*35-Px allele over HLA- B*35-PY (111). Then, they showed that allostimulatory functions of DCs were inhibited by LILRB2 interacting with soluble HLA class I found predominantly in HIV-1–positive plasma (105). The binding affinity to LILRB2 seems to depend on the epitope specificity as sensitive as a single amino acid mutation, as with the HLA-B2705–restricted HIV-1 Gag KK10 epitope (KRWII**L**GLNK) with an L-to-M amino acid substitution at position 6 (L6M) (106). Moreover, soluble HLA-G significantly upregulated during HIV infection interacts with LILRB2 on conventional DCs (cDCs), thus resulting in their functional impairment (107, 108).

In line with these observations, our team demonstrated enhanced LILRB2 and HLA-I expression on cDCs in the early stages of HIV and simian immunodeficiency virus infection that could be responsible for early cDC dysfunction and failure of the subsequent adaptive immune response to control viral infection (101). By contrast, the LILRB2/HLA-I axis was decreased in cDCs in a chikungunya model of infection characterized by efficient immune responses and rapid control of viral replication (101, 109). This accumulating evidence indicates that the enhanced expression of LILRB2 and its HLA-I ligands in early HIV-1 infection could be a potent way to inhibit cDC functions leading to improper adaptive immune responses and ultimately to disease progression. In agreement with this hypothesis, the strength of the LILRB2–HLA-I interaction, modulated by HLA-I haplotypes or HIV-derived peptide, was directly correlated with the level of cDC dysregulation and disease progression in HIV-infected patients (106, 108).

Besides being involved in cDCs, elevated levels of serological IL-10 in HIV-1–positive patients promoted LILRB2 upregulation in CD14^+^ monocytes *via* the signal transducer and activator of transcription 3 pathway (110).

In contrast to LILRB2, the expression of LILRB1 and LILRB3 was increased in cDCs from elite controllers as compared with HIV-1 progressors or healthy individuals (102). The examination of HIV-1 infected patients revealed an upregulation of LILRB1 on subsets of NK cell subsets (112). Further investigations with an *in vitro* model of NK cells co-cultured with HIV-infected autologous monocyte-derived DCs showed an enhanced capacity of NK cells expressing LILRB1 to control viral replication (113).

Activating LILR members are also emerging relevant actors in HIV infection. LILRA1 and LILRA3 may affect the HIV outcome by binding to HLA-C, known to play a major role in disease control (29, 114). Nevertheless, a recent report linked a 6.7-kb deletion in LILRA3 with increased transmission risk for HIV (115). Moreover, the interaction of LILRA4 with BST2 drives pDC dysregulation in HIV-1 infection characterized by inhibition of type-I IFN production (116). All the progress in the study of LILR function in HIV progression highlights the importance of an equilibrium balance between activating and inhibitory signals to ensure efficient immune responses for preventing HIV viral replication, dissemination and pathogenesis. Yet, further investigations are needed to elucidate the convenient mechanisms at the origin of this LILR dysregulation in HIV infection.

**Figure 4:**
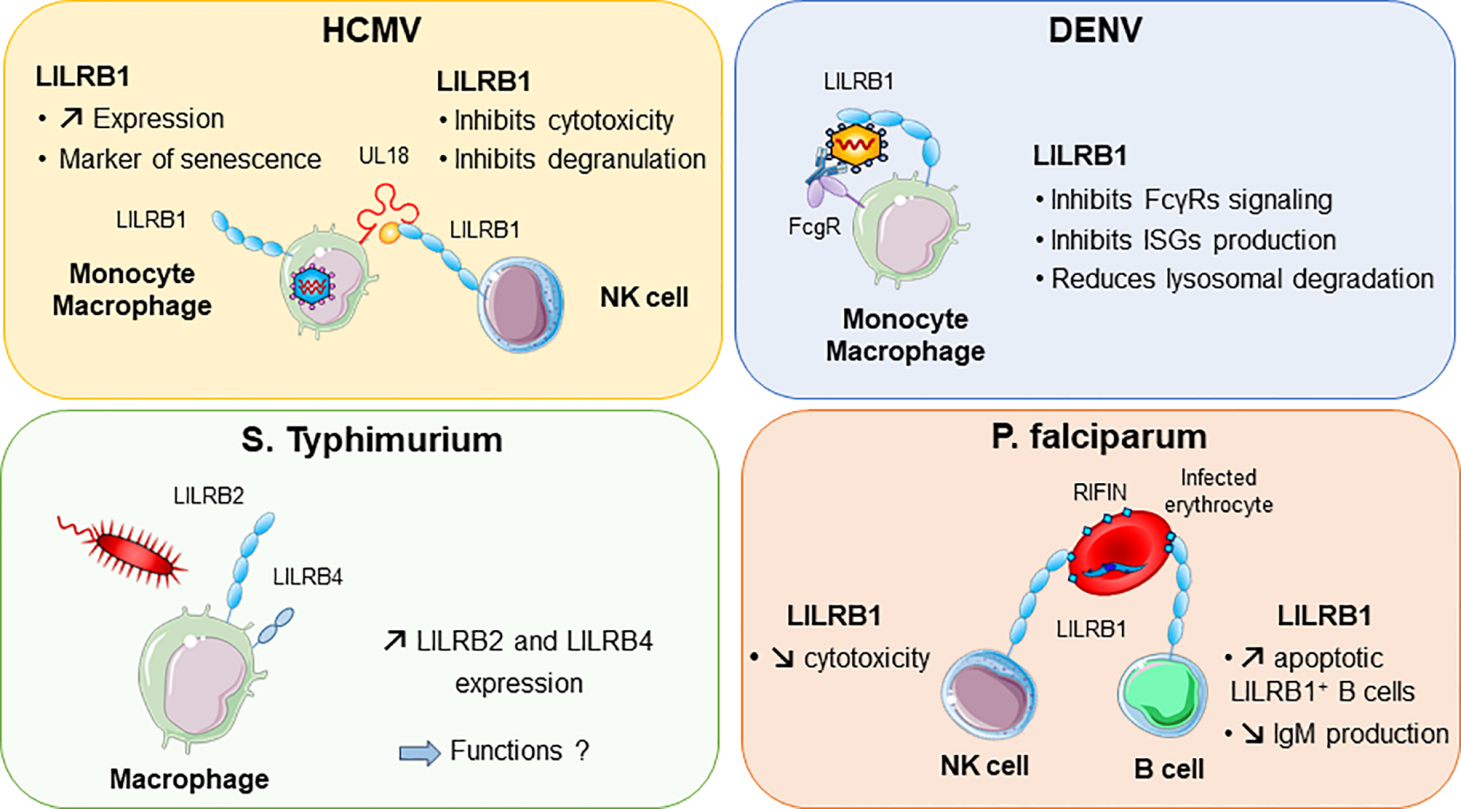
LILRB1 and LILRB2 implication in various infectious diseases. Human cytomegalovirus (HCMV, in purple) produces UL18 protein that binds to LILRB1 with high affinity and regulates NK cell functions. Dengue virus (DENV in blue) can directly bind to LILRB1 expressed by monocytes and macrophages. This interaction inhibits the production of interferon (IFN)-stimulated genes (ISG). In red, *Plasmodium falciparum* produces RIFINs in infected erythrocytes. Binding of RIFINs to LILRB1 inhibits NK cell cytolytic activity and production of IgM by B cells. Exposure of macrophages to *S. typhimurium* bacteria promotes the upregulation of LILRB2 and LILRB4.

Other viruses, including dengue virus and HCMV, were shown to attenuate inflammation and associated immune responses by targeting LILRB1 inhibitory functions (**Figure 4**). In this regard, dengue virus manages to shut down the monocyte antiviral defence program by inhibiting IFN-stimulated gene transcription. This inhibition is mediated by dengue virus ligating to LILRB1 and leading to FcγR-mediated early IFN-blocked gene expression, which results in antibody-dependent enhancement and viral replication (45, 117). The ligand interacting with LILRB1 leading to immune response attenuation during dengue virus infection remains to be characterized. By contrast, HCMV modulates the host immune response by expressing UL18 protein, which binds to LILRB1, thus inhibiting immune effector cells. As described above, UL18 has 1000-fold higher affinity to LILRB1 than host HLA-I ligands (31). This finding highlights the strength of a viral protein such as UL18 to compete with host proteins to evade the host immune response.

### LILRs in Parasite Infection

Intracellular parasites have evolved mechanisms to inhibit and escape the immune response to ensure their survival in the host. *P. falciparum*, the causative pathogen agent of malaria, uses a family of proteins called RIFINs to dampen immune cell functions and impair anti-malarial immunity (**Figure 4**). In the blood, *P. falciparum*-infected erythrocytes express RIFINs; a portion of these molecules can bind LILRB1 expressed on a wide range of immune cells including NK and B cells (118). The engagement of LILRB1 by these RIFINs transduces an inhibitory signal that impairs the cytolytic functions of NK cells and inhibits IgM production by primary B cells (47). Hence, *P. falciparum* escapes the host defence by impairing anti-malarial immunity *via* RIFINs, which mimic the binding mode of the HLA-I host ligands of LILRB1 (47). Similarly, LILRB2 is targeted by RIFIN for immune evasion of *P. falciparum* (48). These findings highlight the importance of further studies on LILRB2 functions in malaria, which could be a potential hit to block disease progression. The parasite *Trypanosoma cruzi* causes Chagas disease. The expression of LILRB1 increases during the differentiation of *T. cruzi*-specific CD4^+^T cells, compromising T-cell functions (119). Moreover, the expression of the LILRB1 ligand HLA-G is increased by *T. brucei gambiense* infection (120). These data suggest a common inhibitory pathway induced by an LILRB1–HLA-G interaction after T*rypanosoma* infection.

### LILRs in Bacterial Infection

A modification of the LILRB expression profile was found in several bacterial infections. Moreover, some bacteria were shown to directly bind some LILRs, which underlies their potential implication in the shaping of inflammation and regulation of immune responses in bacterial infection. LILRB2 and LILRB4 were upregulated in response to *Salmonella* infection, which highlights a potential role of these inhibitory receptors in balancing the inflammatory response after invasion by this pathogen (**Figure 4**) (121). Mycobacteria challenge induces LILRB5 expression in T cells. The purpose of LILRB5 induction could be to initiate an inflammation resolution program by dampening T-cell activation. However, it could also constitute a mechanism induced by *M. tuberculosis* to escape immune recognition (54). Another important emerging role of LILRs is their ability to mediate control of TLR activity in certain bacterial infections. Investigations *in vitro* and in a mouse model provided clues about the ability of LILR and murine PIR-B (a human LILRB distant homolog) to bind in conjunction *S. aureus* and TLR2, thus promoting release of inhibitory cytokines such as IL-10 (46). This LILR-mediated TLR2 inhibition is associated with the Th2 response and impaired DC maturation (122). Moreover, *in vitro* studies indicated that LILRB1 and LILRB3 bind to *S. aureus*, but the consequences of these interactions remain to be characterized (46).

Studies of *Mycobacterium leprae* infection identified the overexpression of LILRA2 in skin lesions of leprosy patients (123). This LILRA2 overexpression exerts innate host defence modifications by driving monocytes to produce IL-10 instead of IL-12 and by inhibiting antimicrobial activity in response to mycobacterial TLR2/1 ligands (123, 124).

However, LILRA2 can promote neutrophil and monocyte activation to inhibit bacterial growth. Indeed, LILRA2 can recognize degraded antibodies generated by bacterial and fungal proteases including *Mycoplasma hyorhinis*, *Legionella pneumophila*, *Streptococcus pneumonia* and *Candida albicans* (55, 92). The engagement of LILRA2 by cleaved Igs stimulates ROS production by neutrophils and inhibits bacterial multiplication in monocytes (55). Hence, in this case, LILRA2 serves as a microbial sensor that promotes the early onset of innate immunity.

### LILRs in Sepsis

Increasing evidence has indicated an interplay between LILRB2 modulation and the host response to sepsis initiated by exacerbated inflammation and followed by immunosuppressive responses. In this regard, transcriptomic analysis showed a significant downregulation of LILRB2 in the very early phase of sepsis that is associated with increased risk of death (125). Another study demonstrated an increased expression of LILRB2 in monocytes during severe sepsis. These LILRB2^high^ monocytes showed low IL-12 but high IL-10 production in response to endotoxin stimulation (126). Moreover, neutrophils from septic patients were unable to upregulate LILRB2 as compared with neutrophils from healthy donors (75). This failure of LILRB2 upregulation on neutrophils may correspond to the immunoparalysis state observed in sepsis patients. LILRB3, LILRB4, LILRA3 and LILRA5 were found highly upregulated in neonatal sepsis (127). This dysregulated profile of LILRs was proposed to interfere with APC activity and convert effective T cells into suppressive T cells. A recent study showed increased LILRB3 expression on macrophages associated with an immunosuppressive role in septic patients. Accordingly, the silencing of LILRB3 was accompanied by bacterial killing by macrophages along with enhanced ROS and cathelicidin production (128). All these data point to the contribution of LILRs to the dysregulation of immune and inflammatory responses induced during septic shock.

## Beyond Infections: LILRs in Inflammation and Autoimmunity

Although LILRs have in certain circumstances a beneficial outcome during infections, they may also disrupt the immunologic balance, resulting in persistent inflammation and autoimmunity. In this regard, the key role of LILRs in immune homeostasis places them at the crossroads of tolerance and autoimmune disorders. Quite a few autoimmune diseases are associated with an aberrant expression of LILRs. LILRB2, LILRB3 and LILRA2 were highly upregulated in synovial tissues from rheumatoid arthritis (RA) patients. The engagement of LILRA2 induced an exacerbated production of TNF-a by macrophages illustrating the potential role of LILRs in the induction of inflammatory processes associated with RA (129). In addition, Kollnberger et al. provided evidence for the contribution of LILRB2 to joint inflammation and disease pathogenesis by HLA-B27 binding in spondylarthritides (130). LILRA3 is also associated with RA severity in early disease (131). Systemic lupus erythematosus (SLE) is another autoimmune condition with modified expression of different LILR members. A recent study reported that LILRA3 amount in both serum and CD14^+^ monocytes were significantly elevated and positively correlated with disease severity in SLE patients (132). Data from humanized mice suggest LILRA3 as a promoter of excessive expression of T follicular helper cells and B cells, which primes the induction and maintenance of plasma cell differentiation and autoantibody production (133). Of note, enhanced expression of LILRB1 was achieved after *ex vivo* IL-10 stimulation of DCs isolated from SLE patients (134). In addition, plasmablasts and plasma cells from SLE patients showed increased expression of LILRB4. Intriguingly, these LILRB4^high^ cells contained abundant anti-double-stranded DNA Ig V_H_ transcripts, which suggests a contradictory role of this inhibitory receptor as a pathogenic marker. Hence, further studies are needed to elucidate the impact of LILRB4 on SLE pathogenesis (135). Moreover, multiple sclerosis (MS) is considered an autoimmune disease characterized by demyelinating the central nervous system caused in a part by deregulated T-cell response (136). MS patients showed aberrant expression of LILRA3 and LILRB1 that was concomitant with disease severity. Serum levels of LILRA3 were associated with clinical subtype of MS: patients with primary progressive disease had the highest amounts of LILRA3 as compared with those with intermediate progressing disease. In line with this, LILRA3 might play a role in the pathogenesis by promoting chronic inflammation and could be considered a biomarker and indicator of MS severity. Nevertheless, the exact functions of LILRA3 in MS require further exploration (137).

## Targeting LILRs for Next-Generation Therapeutics in Infectious Diseases

The use of immune checkpoint agonists or antagonists for treating certain debilitating human inflammatory diseases has drawn a lot of interest. Particularly, monoclonal antibodies (mAbs) have valuable advantages as pharmacological immune modulators and have been used successfully as immune checkpoint inhibitors both in preclinical and clinical settings, including notably mAbs targeting the CTLA-4, PD-1–PD-L1 axis in chronic infectious diseases (138). Notably, mAbs show high stability and high affinity to their molecules and can be used to activate (agonists) or block (antagonists) the function of the intended target (139). Agonists to LILRA and/or anti‐LILRB antagonists that compete with ligand binding without activating LILRB signaling could promote immune responses against infectious agents. Nonetheless, until now, most preclinical and clinical research has focused on evaluating such therapeutic approaches in the treatment of solid tumors (140, 141). For instance, JTX-8064-101 (Jounce Therapeutics Inc.) is currently in an open-label, dose escalation phase-1 clinical trial (NCT04669899). The trial is designed to evaluate the safety, tolerability, and recommended phase-2 dose of JTX-8064 (anti-LILRB2) alone and in combination with JTX-4014 (anti-PD-1) or pembrolizumab (anti-PD-1) in adults with advanced refractory solid tumors. At the same time, another clinical trial including an anti-LILRB4 antibody (IO-202, Immune-Onc Therapeutics Inc.) is in a clinical phase-1 trial (NCT04372433) to assess its safety and primary effects in regression of solid tumors. Yet, in infectious diseases, although preclinical and clinical research investigated various immune checkpoint targets such as the CTLA-4 and PD-1–PD-L1 axis (138, 142) LILRs as therapeutic targets remain to be properly appraised but represent a strong research premise in intractable infectious diseases. LILR family members provide various possible therapeutic strategies to reverse or enhance the immunity failure for the benefit of patients. As in cancer therapy, next-generation therapeutics in infectious diseases will probably combine an immune checkpoint blockade with a strategy targeting agonists to LILRA and/or antagonists to LILRB receptors. Therefore, clarification and exposition of the fundamental function and mechanism of action of LILRs in infectious diseases is vital for accelerating the development of innovative therapeutics involving LILR agonists and/or antagonists, especially in severe acute or persistent infectious diseases lacking appropriate treatment.

In this regard, plasmodium falciparum, Dengue virus and HCMV in human host are often persistent pathogens taking an advantage in targeting LILRB1 to attenuate immune effector cells and evade immune recognition. The evolution of these pathogens toward the production of their own ligands (e.g. RIFINS or UL18) to trigger LILRB1 inhibitory functions illustrate the important role of this receptor in the control of immune responses. In this context, there is a need to counteract weakened immune responses through LILRB1 inadequate activation that can be otherwise used by pathogens as an efficient mechanism of immune evasion and persistence in the host. Henceforth, therapeutic blockade of LILRB1 with mAbs could exhibit a high potential therapeutically for number of severe infections by thwarting pathogen-derived ligands and thus allowing the host immune responses to resolve the disease and clear the infectious pathogens. Likewise, HIV infection induces dendritic cell dysregulation by exacerbating the LILRB2 inhibitory activity, subsequently leading to inefficient adaptive immune responses and virus replication (105–108, 111). Therefore, the development of preclinical studies using therapeutic anti-LILRB2 mAbs in the setting of HIV infection could provide important information for the design of novel clinical protocols aimed at improving immune responses and blocking disease progression in infected patients.

Up to now, pre-clinical evaluation of LILR-based therapies has been hampered by the lack of clear LILR orthologues in rodents. Given the high expression of LILRs on various myeloid cell subsets including resident tissue macrophages and granulocytes, existing humanized mice models are as well poorly appropriate to characterize either the toxicity or the efficiency of drugs targeting LILRs. Nonetheless, LILR orthologues have been identified in non-human primates and dynamics of LILRB2 and MHC-I expression have been characterized on dendritic cells, monocytes and macrophages in cynomolgus macaque during SIV or Chikungunya infections (101). Given the close phylogeny and similarities of immune responses between human and macaque during infectious diseases, this pre-clinical model could therefore represent a unique opportunity to study LILR-based therapeutic strategies and accelerate their translational in medicine toward humans suffering severe acute and/or persistent infections.

## Conclusion

LILRs are essential in maintaining immune homeostasis and in the shaping of immune responses against pathogens. Certain inhibitory LILRs directly interact with endogenous or microbial ligands, and these interactions provide a large window of opportunities to pathogens to evade the immune response. Conversely, some activating LILRs have evolved to detect and avoid immune evasion mechanisms developed by pathogens. A better understanding of the LILR contribution to host–pathogen interactions using adequate *in vivo* models to study the early phase of the immune response to infection, is essential to develop next-generation therapeutics targeting LILRs and intended to counteract virulence and/or persistence of pathogens in severe acute and chronic infectious diseases lacking appropriate treatments.

## Author Contributions

FA was in charge of the manuscript conception, writing and drafting. BF supervised, corrected and revised the manuscript. SC designed most of the figures and contributed to revise the manuscript. MG contributed in figure legend writing and manuscript editing. AN contributed in manuscript writing and editing. LA-R, NS, FM, and OL contributed in manuscript editing. All authors contributed to the article and approved the submitted version.

## Funding

SC received a Ph.D scholarship from the University Paris Saclay and Sidaction. FM received a Ph.D scholarship from the University Paris Saclay. MG received a Ph.D scholarship from theUniversity Paris Saclay and from ANRS. This manuscript was supported by ANRS, the French government’s Investissements d’Avenir program ANR-11-INBS-0008, which funded the Infectious Disease Models and Innovative Therapies infrastructure (IDMIT, Fontenay-aux-Roses, France), and ANR-10-EQPX-02–01, which funded the FlowCyTech facility (IDMIT, Fontenay-aux-Roses, France).

## Conflict of Interest

The authors declare that the research was conducted in the absence of any commercial or financial relationships that could be construed as a potential conflict of interest.

## Publisher’s Note

All claims expressed in this article are solely those of the authors and do not necessarily represent those of their affiliated organizations, or those of the publisher, the editors and the reviewers. Any product that may be evaluated in this article, or claim that may be made by its manufacturer, is not guaranteed or endorsed by the publisher.
